# Effect of Metformin on Renal Function After Primary Percutaneous Coronary Intervention in Patients Without Diabetes Presenting with ST-elevation Myocardial Infarction: Data from the GIPS-III Trial

**DOI:** 10.1007/s10557-015-6618-1

**Published:** 2015-10-20

**Authors:** Rene A. Posma, Chris P. H. Lexis, Erik Lipsic, Maarten W. N. Nijsten, Kevin Damman, Daan J. Touw, Dirk Jan van Veldhuisen, Pim van der Harst, Iwan C. C. van der Horst

**Affiliations:** Department of Critical Care, University of Groningen, University Medical Center Groningen, Hanzeplein 1, P.O. Box 30.001, 9700 RB Groningen, The Netherlands; Department of Cardiology, University of Groningen, University Medical Center Groningen, Groningen, The Netherlands; Department of Clinical Pharmacy and Pharmacology, University of Groningen, University Medical Center Groningen, Groningen, The Netherlands

**Keywords:** Metformin, Myocardial infarction, Renal function, Acute kidney injury

## Abstract

**Purpose:**

The association between metformin use and renal function needs further to be elucidated since data are insufficient whether metformin affects renal function in higher risk populations such as after ST-elevation myocardial infarction (STEMI).

**Methods:**

We studied 379 patients included in the GIPS-III trial in which patients without diabetes or renal dysfunction, who underwent primary percutaneous coronary interventions (PCI) for STEMI, were randomized to metformin 500 mg or placebo twice daily for four months. At baseline and at seven scheduled visits up to four months after PCI, estimated glomerular filtration rate (eGFR) was determined (2582 values). Contrast-induced acute kidney injury (CI-AKI) was defined as an increase in serum creatinine of ≥0.3 mg/dl or 25 % rise within 48 h after PCI.

**Results:**

At all visits, the mean eGFR was similar in patients randomized to metformin or placebo. Over the four month period, mixed-effect repeated-measures model analysis showed a least-squares mean ± standard error change in eGFR of -5.9 ± 0.8 ml/min/1.73 m^2^ in the metformin group and −7.1 ± 0.8 ml/min/1.73 m^2^ in the control group (*P* = 0.27 for overall interaction). The incidence of CI-AKI was 14.8 %; 29 (15.2 %) patients in the metformin group versus 27 (14.4 %) controls (*P* = 0.89). After adjustment for covariates, metformin treatment was not associated with CI-AKI (odds ratio: 0.96, 95%CI 0.52 − 1.75, *P* = 0.88).

**Conclusion:**

We conclude that initiation of metformin shortly after primary PCI has no adverse effect on renal function in patients without diabetes or prior renal impairment, further providing evidence of the safety of metformin use after myocardial infarction and subsequent contrast exposure.

**Electronic supplementary material:**

The online version of this article (doi:10.1007/s10557-015-6618-1) contains supplementary material, which is available to authorized users.

## Introduction

Renal function may decline after primary percutaneous coronary interventions (PCI) for ST-segment elevation myocardial infarction (STEMI) as a result from impaired cardiac function, initiation of pharmacotherapy, and contrast-induced nephropathy. Contrast-induced acute kidney injury (CI-AKI) is observed in approximately 15 % of the patients undergoing coronary interventions for STEMI and is associated with increased short- and long-term morbidity and mortality [1–7]. Although the exact pathophysiology is incompletely understood, changes in renal circulation and subsequent tubular damage are suggested to play a pivotal role [1, 2].

The risk of renal dysfunction after PCI might be increased by the extent of the underlying chronic and acute disease and the use of co-medication, including the antihyperglycemic agent metformin, which is used by millions of patients worldwide for the treatment of type 2 diabetes [8–10]. However, metformin is also associated with a lower risk of decline in renal function in a general population of patients with type 2 diabetes [11–13]. This is of relevance since patients with renal insufficiency are at increased risk of developing metformin-associated lactic acidosis [10]. Metformin is therefore often considered contraindicated since contrast agents may precipitate acute kidney injury, thus allowing metformin and its metabolites to accumulate and lead to lactic acidosis [14–16]. Because data are insufficient regarding the effect of metformin on renal function in higher risk populations, such as after STEMI, we examined whether metformin itself could adversely affect renal function and consequently increase the risk of developing lactic acidosis.

To determine whether metformin treatment initiated shortly after myocardial infarction and subsequent iodinated contrast exposure adversely affects renal function in patients without known diabetes and without pre-existing renal dysfunction, we assessed renal function and the incidence of CI-AKI as a substudy of the Glycometabolic Intervention as Adjunct to Primary Percutaneous Coronary Intervention in ST-Segment Elevation Myocardial Infarction (GIPS) III trial [17].

## Methods

### Trial Design

The design and main results of the GIPS-III trial have been published previously [17, 18]. Briefly, between January 2011 and May 2013, the double-blind GIPS-III trial randomized 380 adult patients who underwent primary PCI for STEMI to receive a 4 month regimen with either 500 mg twice daily metformin (191 patients) or a matching placebo (189 patients). The study medication was initiated as soon as possible after PCI, with the aim of administering the first dose within 3 h after PCI. Exclusion criteria included previous myocardial infarction, known diabetes, the need to perform coronary artery bypass graft surgery, severe renal dysfunction (creatinine >2 mg/dl [177 μmol/L] at baseline), and contraindications for (later) magnetic resonance imaging. During primary PCI, the low-osmolar contrast agents Ioxaglate and Iobitridol were used. Secondary treatment was according to current guidelines [20]. Study medication was discontinued when subjects did develop severe renal dysfunction (defined as creatinine >2 mg/dl [177 μmol/L], or an estimated glomerular filtration rate [eGFR] <30 ml/min/1.73 m^2^) [18].

The study protocol was in accordance with the Declaration of Helsinki and was approved by the institutional review board and national regulatory authorities (METc 2010.077). Informed consent was obtained from all participants. This trial was registered at ClinicalTrials.gov (Trial identifier: NCT01217307).

### Assessment of Renal Function

The measurements of serum creatinine concentrations were planned at baseline, 6 h, 12 h, 24 h, 48 h, 2 weeks, 6 weeks, and 4 months after PCI [18]. For patients who were returned to their referring center after PCI, creatinine concentration measurements during hospital admission and up to four months were obtained. Estimated glomerular filtration rate (eGFR) was calculated using the Chronic Kidney Disease Epidemiology Collaboration study equation, as has been suggested to estimate the GFR more accurate than the simplified Modification of Diet in Renal Disease (sMDRD) formula in patients without severe renal impairment [20, 21]. CI-AKI was defined as an increase in serum creatinine of ≥0.3 mg/dL (27 μmol/L), or a 25 % relative rise in creatinine, within 48 h after the start of the PCI procedure [1].

### Statistical Analyses

Normally distributed continuous data are presented as means with standard errors (SE), unless stated otherwise, and differences were assessed using the Student’s t-test. If normality could not be assumed, the data are presented as medians with interquartile range (IQR), and differences were assessed using the Mann-Whitney U test. Categorical data are presented as frequencies with percentages, and differences were assessed using the chi-square or the Fisher exact test, when appropriate.

To estimate and compare the effect of metformin on renal function, mixed-effects repeated measures analysis with random slope and intercept was performed. This type of analysis operates using estimation techniques that allow for incomplete data. Age, gender, baseline N-terminal pro–B-type natriuretic peptide (NT-proBNP) concentration, and myocardial blush grade after PCI were, based on the statistical plan of the main study, considered as covariables in relation to the renal function [17, 18]. Only significant covariables were used as fixed effects in the final multivariate model. Individual patients were considered as a random effect. The covariance matrix of residuals used in the model was unstructured. To determine the effect of metformin on renal function within the subset of CI-AKI patients, we used a mixed-effects repeated measures model similar to the primary analysis, but now including a three way interaction variable including time, the occurrence of CI-AKI, and treatment allocation.

The independent predictors of CI-AKI were identified by a backward-stepwise logistic regression model using an entry level of significance of 0.1. Randomization to metformin or placebo, gender, age, contrast dose, eGFR at baseline, and anemia (Hb < 13.7 mg/dl [<8.5 mmol/L] in men and Hb < 12.1 mg/dl [<7.5 mmol/L] in women) were previously associated with the development of CI-AKI and were therefore forced in the multivariate analysis in addition to risk factors found in the univariate analysis [4, 8, 9, 22].

All reported *P-*values are 2-sided and *P* < 0.05 was considered statistically significant, except for interactions in which *P* < 0.10 was considered significant. Statistical analyses were performed by using SPSS Statistics for Windows, Version 22 (IBM, Armonk, NY) and Stata version 12.0 (Stata Corp., College Station, TX). The complete methods can be reviewed in Online Resource 1.

## Results

### Study Population

**Fig. 1:**
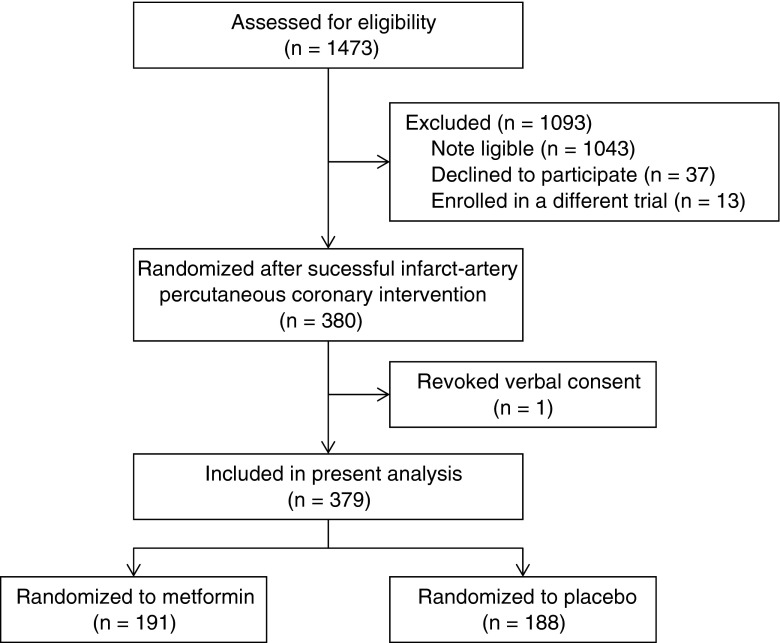
Flow of patients through the glycometabolic intervention as adjunct to primary coronary intervention in the ST-segment elevation myocardial infarction (GIPS-III) trial

A total of 380 patients were randomized in the GIPS-III trial; 191 patients were allocated to the metformin treatment and 189 patients to placebo. Of those, 379 patients were included in the present analysis (one patient, randomized to placebo, withdrew informed consent), as depicted in Figure 1. A total of 2582 serum creatinine measurements at the eight fixed time points were available, with a median (range) of 7 (3–8) measurements per patient. Serum creatinine concentration was measured at baseline in 379 (100 %), at 6 h in 372 (98 %), at 12 h in 365 (96 %), at 24 h in 297 (78 %), at 48 h in 154 (41 %), at 2 weeks in 346 (91 %), at 6 weeks in 336 (89 %), and at 4 months in 333 (88 %) patients, respectively. The main reason for missing creatinine values at 48 h was early discharge of low-risk patients (i.e., younger age, smaller myocardial infarction size, better post-procedure myocardial perfusion and left ventricular function, and no history of hypertension) and was not related to treatment allocation (109 [57.1 %] of metformin vs. 116 [61.7 %] of placebo treated patients, *P* = 0.40), which is displayed in Online Resource 2.

Baseline characteristics were similar in both treatment groups (Online Resource 3). The median duration until administration of the first treatment dose after coronary intervention was similar in the metformin group (102 min [IQR 81–133]) and the control group (100 min [78–134], *P* = 0.26). The median duration of exposure to the study medication was also similar in the metformin group (124 days [IQR 119–125]) and the control group (124 days [IQR 120–125], *P* = 0.14) [17].

### Effect of Metformin on Renal Function

In the mixed-effects repeated-measures model analysis, significant associations were found for age and baseline NT-proBNP concentration, but not the overall interaction of time and allocated treatment (*P* = 0.27). From baseline up to four months after PCI, the least-squares mean ± SE change in eGFR was −6.5 ± 0.8 ml/min/1.73 m^2^ in the overall population. In none of the patients the study medication was discontinued due to severe renal dysfunction.

**Fig. 2:**
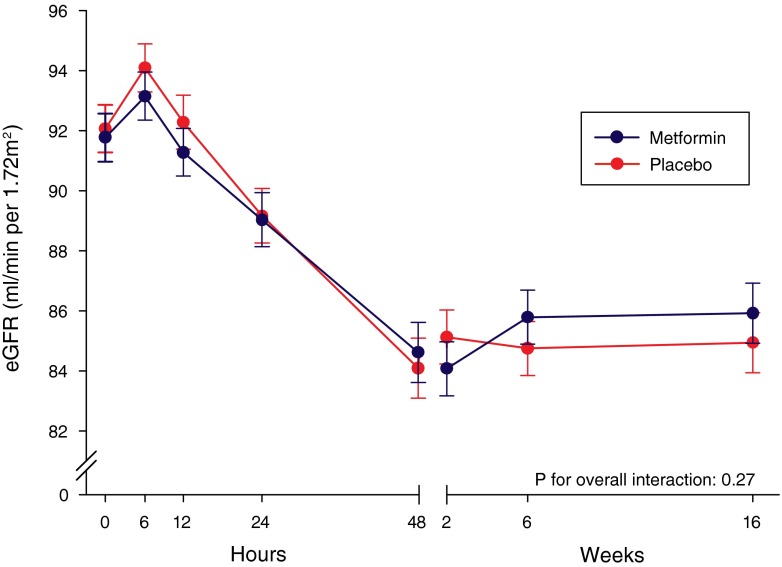
Presented are least-squares means ± standard error from the mixed-effects repeated measurements model with a random intercept and slope. Individual patients were considered as random effects and the following were fixed effects: age, baseline N-terminal pro–B-type natriuretic peptide concentration, and treatment allocation. We assumed an unstructured covariance structure among serial estimated glomerular filtration rate values (eGFR). No significant difference was observed for the overall interaction of time and allocated treatment (*P* = 0.27)

Figure 2 displays the trends of adjusted eGFR over time in patients randomized to metformin or placebo. In the first 48 h, patients in both treatment groups experienced a rapid decrease in mean eGFR of −7.2 ± 0.8 ml/min/1.73 m^2^ in the metformin group and −8.0 ± 0.8 ml/min/1.73 m^2^ in the control group. After 48 h, renal function slightly improved in both groups, resulting in a mean overall decrease in eGFR of −5.9 ± 0.8 ml/min/1.73 m^2^ in the metformin group and −7.1 ± 0.8 ml/min/1.73 m^2^ in the control group from baseline to four months. During hospitalization and at all scheduled visits, no significant differences in adjusted eGFR were observed between the two treatment groups (Online Resource 4).

During hospitalization and at all scheduled visits up to four months after PCI, the unadjusted mean eGFR did not significantly differ in patients randomized to metformin or placebo (Online Resource 5 and 6). When the sMDRD formula was used to estimate the glomerular filtration rate, no significant differences in renal function between patients randomized to metformin or placebo were found during hospitalization and all scheduled visits (Online Resource 6) [23]. A similar observation was made for serum creatinine concentrations (Online Resource 6).

### Contrast-Induced Acute Kidney Injury

CI-AKI was observed in 56 (14.8 %) patients; if CI-AKI was defined as an increase in 0.5 mg/dl, 55 (14.5 %) patients developed CI-AKI. The clinical- and procedural characteristics, cardiovascular history, and laboratory values at baseline in patients who did and who did not develop CI-AKI are presented in Table 1. Patients who developed CI-AKI had a significantly longer ischemic time, a longer coronary intervention procedure, a higher baseline eGFR, a higher baseline concentration of creatine kinase (CK), the myocardial band of CK, and NT-proBNP. The CI-AKI group more frequently used calcium channel blockers before admission, and more frequently received coumarin derivatives and mineralocorticoid receptor antagonists (MRA) during hospitalization (Table 2 and Online Resource 7). The incidence of CI-AKI was similar in the metformin group (29 [15.2 %] patients) and the control group (27 [14.4 %] patients, *P* = 0.89).

**Table 1:** Characteristics of patients who did and did not develop contrast-induced acute kidney injury

	No. (%)			
Characteristic	Total (*n* = 379)	CI-AKI (*n* = 56)	No CI-AKI (*n* = 323)	*P*-value
Metformin as study drug	191 (50.4)	29 (51.8)	162 (50.2)	0.89
Age at randomization, mean (SD), years	58.8 (11.6)	60.6 (11.6)	58.5 (12.1)	0.21
Women	95 (25.1)	19 (33.9)	76 (23.5)	0.13
Body mass index^a^, mean (SD), kg/m^2^	27.0 (3.8)	26.6 (3.5)	27.0 (3.9)	0.41
Race/ethnicity				0.67
White	365 (96.3)	54 (96.4)	311 (96.3)	
Asian	10 (2.6)	1 (1.8)	9 (2.8)	
Black	4 (1.1)	1 (1.8)	3 (0.9)	
Cardiovascular related history				
Hypertension	112 (29.6)	20 (35.7)	92 (28.5)	0.27
Dyslipidemia	239 (63.1)	40 (71.4)	199 (61.6)	0.18
Current smoking	209 (55.1)	31 (55.4)	178 (55.1)	0.97
Stroke	3 (0.8)	0	3 (0.9)	1.00
Peripheral artery disease	0	0	0	
Previous PCI	4 (1.1)	0	4 (1.2)	1.00
Blood pressure, mean (SD), mmHg				
Systolic	134 (23)	136 (24)	134 (23)	0.59
Diastolic	84 (15)	86 (14)	84 (15)	0.46
Heart rate, mean (SD), beats/min	76 (16)	77 (15)	76 (17)	0.59
Anemia^b^	76 (20.1)	9 (16.1)	67 (20.7)	0.48
Ischemia time, median (IQR), min	161 (109–250)	218 (150–339)	150 (107–238)	<0.01
Single vessel disease	258 (68.1)	41 (73.2)	217 (67.2)	0.44
Infarct related artery				
Left anterior descending coronary artery	146 (38.5)	24 (42.9)	122 (37.8)	
Left circumflex coronary artery	64 (16.9)	11 (19.6)	53 (16.4)	
Right coronary artery	169 (44.6)	21 (37.5)	148 (45.8)	
Left main	0	0	0	
Infarct related artery TIMI flow				
Pre-intervention grade				0.06
0	208 (54.9)	37 (66.1)	171 (52.9)	
1	27 (7.1)	5 (8.9)	22 (6.8)	
2	66 (17.4)	5 (8.9)	61 (18.9)	
3	78 (20.6)	9 (16.1)	69 (21.4)	
Post-intervention grade				0.61
2	34 (9.0)	6 (10.7)	28 (8.7)	
3	345 (91.0)	50 (89.3)	295 (91.3)	
Myocardial blush grade				0.29
0	10 (2.6)	2 (3.6)	8 (2.5)	
1	29 (7.7)	6 (10.7)	23 (7.2)	
2	74 (19.5)	12 (21.4)	62 (19.4)	
3	263 (69.4)	36 (64.3)	227 (70.9)	
Procedural characteristics				
Use of Ioxaglate^c^	373 (99.5)	56 (100)	317 (99.4)	1.00
Contrast dose, median (IQR), ml	150 (120–180)	150 (120–200)	140 (120–180)	0.06
Length of procedure, median (IQR), min	31 (22–42)	36 (25–47)	30 (22–40)	0.04
Radiation exposure, median (IQR), μGy/m^2^	5130 (2944–7646)	4791 (3643–7857)	5132 (2825–7645)	0.60
Laboratory values at admission				
Creatinine, mean (SD), μmol/L	73 (15)	65 (15)	75 (15)	<0.01
eGFR^d^, mean (SD), ml/min/1.73 m^2^	92 (16)	96 (15)	91 (16)	0.02
CK, median (IQR), U/L	129 (83–210)	156 (94–320)	125 (82–193)	0.03
Myocardial band of CK, median (IQR), U/L	16 (13–25)	20 (14–42)	16 (12–23)	<0.01
NT-proBNP, median (IQR), ng/L	81 (40–200)	126 (65–309)	75 (37–180)	<0.01
Glucose, median (IQR), mmol/L	8.2 (7.0–9.6)	8.0 (7.3–9.5)	8.4 (7.0–9.6)	0.88
HbA_1C_, median (IQR), %	5.8 (5.6–6.0)	5.8 (5.7–6.0)	5.8 (5.6–6.0)	0.18
Hb, mean (SD), mmol/L	8.9 (0.8)	8.9 (0.8)	8.9 (0.8)	0.43
AUC from baseline up to 24 h				
CK, median (IQR)	7.1 × 10^7^ (3.0 × 10^7^–15.1 × 10^7^)	10.7 × 10^7^ (3.3 × 10^7^–19.5 × 10^7^)	6.6 × 10^7^ (2.9 × 10^7^–14.1 × 10^7^)	0.06
Myocardial band of CK, median (IQR)	8.2 × 10^6^ (3.4 × 10^6^–15.8 × 10^6^)	10.7 × 10^6^ (3.6 × 10^6^ –22.4 × 10^6^)	7.8 × 10^6^ (3.4 × 10^6^–14.8 × 10^6^)	0.09

*CI-AKI* contrast-induced acute kidney injury, *SD* standard deviation, *PCI* percutaneous coronary intervention, *IQR* interquartile range, *TIMI* thrombolysis in myocardial infarction, *CAG* coronary arteriography, *eGFR* estimated glomerular filtration rate, *CK* creatine kinase, *NT-proBNP* N-terminal pro–B-type natriuretic peptide, *HbA*_*1C*_ glycated hemoglobin, *Hb* hemoglobin, *AUC* area under the curve

^a^Calculated as weight in kilograms, divided by lenght in meters squared.

^b^Defined as Hb < 13.7 mg/dl (<8.5 mmol/L) in men and Hb < 12.1 mg/dl (<7.5 mmol/L) in women.

^c^Type of contrast agent was known for 375 (98.9 %) patients

^d^Calculated using the Chronic Kidney Disease Epidemiology Collaboration study equation

**Table 2:** Medical therapy initiated during hospitalization

Drug category	Total (*n* = 379)	CI-AKI (*n* = 56)	No CI-AKI (*n* = 323)	*P*-value
Aspirin	341 (90.0)	48 (85.7)	293 (90.7)	0.24
Coumarin derivative	19 (5.0)	7 (12.5)	12 (3.7)	0.01
Thienopyridines	377 (99.5)	56 (100)	321 (99.4)	1.00
Clopidogrel	268 (70.7)	37 (66.1)	231 (71.5)	0.43
Prasugrel	4 (1.1)	2 (3.6)	2 (0.6)	0.11
Ticagrelor	105 (27.7)	17 (30.4)	88 (27.2)	0.63
ACE-inhibitor or ARB	263 (69.4)	41 (73.2)	222 (68.7)	0.54
Beta-blocker	323 (85.2)	51 (91.1)	272 (84.2)	0.22
Calcium-channel blocker	9 (2.4)	0	9 (2.8)	0.37
Mineralocorticoid receptor antagonist	38 (10.0)	13 (23.2)	25 (7.7)	0.01
Other diuretic	5 (1.3)	1 (1.8)	4 (1.2)	0.55
Statin	343 (90.5)	48 (85.7)	295 (91.3)	0.21
Insulin^a^	5 (1.3)	1 (1.8)	4 (1.2)	0.55
Oral antihyperglycemic agent^a^	4 (1.1)	2 (3.6)	2 (0.6)	0.11

Values are expressed as n (%). Medical therapy initiated during hospitalization until hospital discharge for either discharge home or transfer to a referring hospital. *CI-AKI* contrast-induced acute kidney injury, *ARB* angiotensin-receptor blocker

^a^In addition to the study medication

Multivariate logistic regression analysis identified the initiation of a MRA during hospitalization as the strongest predictor of CI-AKI (odds ratio (OR): 3.30, 95%CI 1.51–7.23, *P* < 0.01), as presented in Table 3. In total, MRAs were started in 38 (10.0 %) patients during hospitalization based on clinical indication. At baseline, an increase in age (per 5 years), eGFR (per 5 ml/min/1.72 m2), and logarithmic transformed NT-proBNP concentration were also associated with an increased risk for CI-AKI. Randomization to metformin was not a predictor of CI-AKI (OR: 0.96, 95%CI 0.52–1.75, *P* = 0.88). When included in the multivariate analysis, contrast dose was not associated with the development of CI-AKI (OR: 1.01 per 10 ml increase, 95%CI 0.98–1.03, *P* = 0.60).

**Table 3:** Predictors for contrast-induced acute kidney injury

	Univariate		Multivariate	
Characteristic	OR (95%CI)	*P-value*	OR (95%CI)	*P-value*
Randomization to metformin	1.07 (0.61–1.88)	0.82	0.96 (0.52–1.75)	0.88
Age (per 5 years increase)	1.08 (0.95–1.21)	0.24	1.31 (1.14–1.55)	<0.01
Female sex	1.67 (0.91–3.07)	0.10		
Ischemia time (per 5 mins increase)	1.01 (1.00–1.02)	<0.01		
Anemia^a^	0.73 (0.34–1.57)	0.42		
Pre-intervention TIMI-flow	0.79 (0.62–1.01)	0.06		
Contrast dose (per 5 ml increase)	1.02 (1.00–1.05)	0.07		
Length of procedure (per 5 mins increase)	1.05 (0.99–1.13)	0.13		
eGFR^b^ (per 5 ml/min/1.72 m^2^ increase)	1.12 (1.01–1.24)	0.03	1.33 (1.14–1.55)	<0.01
CK (per 10 U/L increase)	1.01 (1.00–1.02)	<0.01		
Myocardial band of CK (per 5 U/L increase)	1.03 (1.01–1.06)	<0.01		
NT-proBNP^c^	2.08 (1.28–3.38)	<0.01	1.91 (1.10–3.32)	0.02
Initiation of a coumarin derivative during hospitalization^d^	3.70 (1.39–9.86)	<0.01		
Initiation of a MRA during hospitalization^e^	3.60 (1.72–7.57)	<0.01	3.30 (1.51–7.23)	<0.01

When included in the multivariate analysis, contrast dose was not associated with the development of contrast-induced acute kidney injury (OR: 1.01 per 10 ml increase, 95%CI 0.98–1.03, *P* = 0.60). *OR* odds ratio, *95%CI* 95 % confidence interval, *TIMI* thrombolysis in myocardial infarction, *eGFR* estimated glomerular filtration rate, *CK* creatine kinase, *NT-proBNP* N-terminal pro–B-type natriuretic peptide

^a^Defined as Hb <13.7 mg/dl (<8.5 mmol/L) in men and Hb <12.1 mg/dl (<7.5 mmol/L) in women

^b^Calculated using the Chronic Kidney Disease Epidemiology Collaboration study equation

^c^Log transformed

^d^In total, coumarine derivatives were initiated in 19 (5.0 %) patients during hospitalization on clinical indication

^e^In total, mineralocorticoid receptor antagonists were initiated in 38 (10.0 %) patients during hospitalization on clinical indication

Mixed-effects repeated measures model analysis showed no significant interaction between randomized treatment, CI-AKI, and time with respect to the changes in eGFR (P for interaction = 0.14). Online Resource 8 displays the trends of adjusted eGFR over time in patients with and without CI-AKI who were randomized to metformin or placebo. There was an expected overall interaction between the occurrence of CI-AKI and the change in eGFR over the first 48 h (*P* = 0.021), but there was no difference between randomized treatments (*P* = 0.13). At the end of follow up, eGFR did not differ between patients on placebo or metformin, neither in the CI-AKI group (*P* = 0.34) nor the no CI-AKI group (*P* = 0.66).

## Discussion

In this predefined sub-analysis of the GIPS-III trial, changes in renal function after primary PCI for STEMI were similar in patients randomized to metformin or placebo.

Overall, eGFR declined during the 4 months of follow-up. Several factors might influence this decrease in eGFR, such as a reduction in left ventricular function after myocardial infarction and subsequent medical treatment, including the start of ACE-inhibitors, angiotensin receptor blockers, and MRAs, which are known to be associated with a decrease in eGFR [1, 2, 24–26]. However, randomization to metformin treatment had no effect on decrease in renal function compared to placebo. In addition, no difference was observed in the development of CI-AKI. To our knowledge, this is the first placebo controlled study providing data suggesting that initiation of metformin treatment early after primary PCI is safe in patients without pre-existent renal dysfunction, which is in line with animal- and studies suggesting metformin does not adversely affect renal function or might even be protective by reducing renal tubular damage [11–13, 27–29].

In our population, a higher incidence of CI-AKI was observed when MRAs were initiated during hospitalization. However, this association might be biased by the indication of MRAs. The initiation of a MRA during hospitalization is limited to patients with signs of heart failure and depressed left ventricular function after STEMI [19]. Moreover, the initiation of a MRA is associated with a steeper decline in renal function in patients with heart failure after myocardial infarction [26].

Because of the exclusion of patients with known diabetes, prior myocardial infarction, or pre-existing renal impairment, the included patients had favorable baseline characteristics and had a lower risk to develop renal dysfunction in comparison to STEMI patients with these risk factors. Furthermore, the median contrast dose of 150 ml was considerably lower than previous studies evaluating patients who underwent PCI for myocardial infarction [4, 6, 8, 22]. In our study, the incidence of CI-AKI was around 15 % after 48 h, which is similar to studies with a more heterogeneous patient population [4–6, 22]. Possibly, we observed a comparable incidence of CI-AKI in our population because of the lower baseline creatinine concentration and consequently, due to regression to the mean, a higher chance of meeting the CI-AKI criteria [5, 6, 8, 22]. Therefore current CI-AKI criteria may not be the best measure to identify renal dysfunction in lower risk STEMI populations, such as ours. Also the term CI-AKI might wrongly suggest that the cause of renal function decline is solely due to contrast use, because myocardial ischemia also affects cardiac function and the initiation of pharmacotherapy might affect the estimated renal function parameters as well [25, 30].

Our findings may have an important clinical implication. Due to an increased risk for lactic acidosis, metformin is currently often considered to be contraindicated in patients with mild renal impairment until 48 h after procedures using iodinated contrast agents [14–16]. During the study period, no cases of lactic acidosis (lactate >5 mmol/L and pH <7.35) were observed [17]. We believe that in patients without severe renal impairment, metformin treatment can be started early after the procedure [10, 31, 32]. Since this study only evaluates the effect of metformin initiated shortly after PCI, further investigation is needed to determine the safety or even protective effect of initiation of metformin prior to PCI or continuation of metformin during PCI procedures.

We recognize that there are also limitations to our study. First, metformin treatment was initiated after coronary intervention, with a median delay of 101 min until the first dose of study medication was administered, resulting in an average time between contrast exposure and the achievement of effective plasma levels of approximately 4 h [17]. The lack of beneficial effect of metformin on renal function might also be attributable to the relatively low dose of metformin administered (500 mg twice daily). Second, the doses of contrast agent used during the procedures were lower than in other studies and were not associated with development of CI-AKI after adjustment for covariates [4, 6, 8]. Finally, the GIPS-III study excluded patients with severe renal impairment, defined as a baseline creatinine level of >2 mg/dl (177 μmol/L), which is the patient group that is most prone to developing renal dysfunction [4, 8, 9, 22, 33]. Since the majority of the study population were Caucasian and male, generalizability of the results to females and other races will be partly limited.

We conclude that the initiation of metformin shortly after myocardial infarction and contrast exposure had no adverse effect on renal function and did not influence the development of CI-AKI in patients without diabetes and without prior renal impairment undergoing primary PCI for STEMI. Metformin use in higher risk patients deserves further investigation.

## Electronic supplementary material

ESM 1 (PDF 485 kb)
