# Supplementary material for: Effect of Metformin on Renal Function After Primary Percutaneous Coronary Intervention in Patients Without Diabetes Presenting with ST-elevation Myocardial Infarction: Data from the GIPS-III Trial
Source: Cardiovasc Drugs Ther. 2015 Oct 20;29(5):451–9. doi: 10.1007/s10557-015-6618-1 (PMC4636992; doi:10.1007/s10557-015-6618-1)
Supplement: Supplementary file 1 — (PDF 485 kb) [file 10557_2015_6618_MOESM1_ESM.pdf]

Online Resource 1 – Complete methods

GIPS-III trial

The Glycometabolic Intervention as Adjunct to Primary Percutaneous Coronary Intervention in ST-Segment Elevation Myocardial Infarction (GIPS) III study is a double-blind placebo-controlled randomized clinical trial and was designed to determine whether metformin preserves left ventricular function after ST-segment elevation myocardial infaction (STEMI) in patients without diabetes. The design and main results of the GIPS-III trial has been described in detail previously.[1,2]

All patients admitted to the University Medical Center Groningen between January 1, 2011, and May 26, 2013, via the STEMI protocol were considered eligible for this trial. Inclusion criteria were age older than 18 years, the presence of STEMI and primary percutaneous coronary intervention (PCI) with implantation of at least 1 stent with a diameter of at least 3 mm resulting in Thrombolysis in Myocardial Infarction flow grade 2 or 3 post PCI. Major exclusion criteria were previous myocardial infarction, known diabetes, the need for coronary artery bypass graft surgery, severe renal dysfunction (>2 mg/dl (177 μmol/L)), and standard contraindications for MRI.

On admission, standard laboratory assessment including serum concentrations of creatine kinase (CK), myocardial band of CK, creatinine, N-terminal pro brain natriuretic peptide (NT-proBNP), blood glucose, and glycated hemoglobin was performed. Standard physical examination parameters including blood pressure, heart rate, and body mass index were measured. Coronary angiography was performed using standard techniques. The choice and order of coronary intervention (i.e., thrombus aspiration, balloon angioplasty, or stenting) was left to the discretion of the operator. During the PCI procedure, all patients provided verbal informed consent in the presence of an independent witness.

After arrival in the coronary care unit, patients were randomly assigned in a 1:1 ratio, using block randomization of 6 patients, to a four month treatment with either metformin hydrochloride (500 mg) or a visually matching placebo, both administered twice daily, blinded to patients and investigators. The study medication was started as soon as possible after PCI, with the aim of administering the first dose within 3 hours after PCI.

Following admission to the coronary care unit, patients provided written informed consent. All patients received standard treatment for myocardial infarction. During primary PCI, the low-osmolar contrast agents Ioxaglate and Iobitridol were used. Secondary treatment was according to current guidelines.[3] Patients were offered rehabilitation programs for myocardial infarction and were advised on diet, smoking, and lifestyle changes according to a standardized protocol introduced before study initiation.

Manufacturing and packaging including blinding was performed by Stichting Apotheek Haagse Ziekenhuizen, Den Haag, the Netherlands, according to the Good Manufacturing Practice standards of the European Union. Study drug adherence was assessed by tablet counts at the visits to the outpatient clinic.

Study monitoring, data management, and validation were independently performed at the Trial Coordination Center (University Medical Center Groningen, the Netherlands).

The study protocol was in accordance with the Declaration of Helsinki and was approved by the institutional review board (METC 2010.077, Groningen, the Netherlands) and national regulatory authorities. This trial was registered at clinicaltrials.gov (NCT01217307).

Assessment of renal function

The measurements of serum creatinine concentrations were planned at baseline, 6h, 12h, 24h, 48h, 2 weeks, 6 weeks, and 4 months after PCI.[2] For patients who were returned to their a referring center after PCI, creatinine concentration measurements during hospital admission and up to four months after PCI were obtained. When more than one measurement was available at a specific time point, the measurement that was nearest to the designated time point was used for analysis.

Estimated glomerular filtration rate (eGFR) was calculated using the Chronic Kidney Disease Epidemiology Collaboration study equation[4], as has been suggested to estimate the GFR more accurate than the simplified Modification of Diet in Renal Disease formula in patients without severe renal impairment.[5] Contrast-induced acute kidney injury was defined as an increase in serum creatinine of ≥0.3 mg/dL (27 μmol/L), or a 25% relative rise in creatinine, within 48 hours after the start of the PCI procedure.[6]

Time of lidocaine injection defined the start of the PCI procedure. If unavailable, arrival time at the catheterization lab was used. The application of a closure device defined the end of the procedure. The extent of myocardial damage was estimated by calculating the area under the curve of CK and the myocardial band of CK from baseline until 24 hours after PCI.

Statistical analysis

Normally distributed continuous data are presented as means with standard error, unless stated otherwise, and differences were assessed using the Student's t-test. If normality could not be assumed, the data are presented as medians with interquartile range, and differences were assessed using the Mann-Whitney U test. Categorical data are presented as frequencies with percentages, and differences were assessed using the chi-square or the Fisher exact test, when appropriate.

To estimate and compare the effect of metformin on renal function, mixed-effects repeated measures analysis with random slope and intercept was performed. This type of analysis operates using estimation techniques that allow for incomplete data. Age, gender, baseline NT-proBNP concentration, and myocardial blush grade after PCI were, based on the statistical plan of the main study, considered as covariables in relation to the renal function.[1] Only significant covariables were used as fixed effects in the final multivariate model. Individual patients were considered as a random effect. The covariance matrix of residuals used in the model was unstructured.

The independent predictors of CI-AKI were identified by a backward-stepwise logistic regression model using an entry level of significance of 0.1. Randomization to metformin or placebo, gender, age, contrast dose, eGFR at baseline, and anemia (hemoglobin (Hb) <13.7 mg/dl (<8.5 mmol/L) in men and Hb <12.1 mg/dl (<7.5 mmol/L) in women) were previously associated with the development of CI-AKI and were therefore forced in the multivariate analysis in addition to risk factors found in the univariate analysis.[7-10]

All reported P-values are 2-sided and P<0.05 was considered statistically significant, except for interactions in which P<0.10 was considered significant. Since this study concerns a post-hoc analysis of a previously conducted randomized controlled trial, no sample size calculation was made. Statistical analyses were performed by using SPSS Statistics for Windows, Version 22 (IBM, Armonk, NY) and Stata version 12.0 (Stata Corp., College Station, TX).

References

1. Lexis CP, van der Horst IC, Lipsic E, et al. Effect of metformin on left ventricular function after acute myocardial infarction in patients without diabetes: the GIPS-III randomized clinical trial. JAMA 2014; 311:1526–1535.

2. Lexis CP, van der Horst IC, Lipsic E, et al. Metformin in non-diabetic patients presenting with ST elevation myocardial infarction: rationale and design of the glycometabolic intervention as adjunct to primary percutaneous intervention in ST elevation myocardial infarction (GIPS)-III trial. Cardiovasc Drugs Ther 2012; 26:417-426.

3. Task Force on the management of ST-segment elevation acute myocardial infarction of the European Society of Cardiology (ESC), Steg PG, James SK, et al. ESC Guidelines for the management of acute myocardial infarction in patients presenting with ST-segment elevation. Eur Heart J 2012; 33:2569-2619.

4. Levey AS, Stevens LA, Schmid CH, et al. A new equation to estimate glomerular filtration rate. Ann Intern Med 2009, 150:604-612.

5. Levey AS, Inker LA, Coresh J. GFR estimation: from physiology to public health. Am J Kidney Dis 2014; 63:820-834.

6. McCullough PA. Contrast-induced acute kidney injury. *J Am Coll Cardiol* 2008; 51:1419-1428.

7. Narula A, Mehran R, Weisz G, et al. Contrast-induced acute kidney injury after primary percutaneous coronary intervention: results from the HORIZONS-AMI substudy. *Eur Heart J* 2014; 35:1533-1540.

8. Tziakas D, Chalikias G, Stakos D, et al. Validation of a New Risk Score to Predict Contrast-Induced Nephropathy After Percutaneous Coronary Intervention. *Am J Cardiol* 2014; 113:1487-1493.

9. Tziakas D, Chalikias G, Stakos D, et al. Development of an easily applicable risk score model for contrast-induced nephropathy prediction after percutaneous coronary intervention: a novel approach tailored to current practice. *Int J Cardiol* 2013; 163:46-55.

10. Mehran R, Aymong ED, Nikolsky E, et al. A simple risk score for prediction of contrast-induced nephropathy after percutaneous coronary intervention: development and initial validation. *J Am Coll Cardiol* 2004; 44:1393-1399.

Online Resource 2 – Patients who did and did not have a creatinine measurement available at 48 hours after PCI

Characteristic	No. (%) Measurement available (n = 154)	No. (%) Measurement not available (n = 225)	P-value
Randomization to metformin	82 (53.2)	109 (48.4)	0.40
Age at randomization, mean (SD), years	60.3 (12.6)	57.7 (10.9)	0.03
Women	49 (31.8)	46 (20.4)	0.02
Body mass index*, mean (SD), kg/m^2	27.1 (3.6)	26.9 (4.0)	0.64
Race/ethnicity			
White	149 (96.8)	216 (96.0)	0.19
Asian	2 (1.3)	8 (3.6)	
Black	3 (1.9)	1 (0.4)	
**Cardiovascular related history**			
Hypertension	58 (37.7)	54 (24.0)	<0.01
Dyslipidemia	104 (67.5)	135 (60.0)	0.16
Current smoking	78 (50.6)	131 (58.2)	0.17
Stroke	2 (1.3)	1 (0.4)	0.57
Peripheral artery disease	0	0	1.00
Previous PCI	2 (1.3)	2 (0.9)	1.00
Blood pressure, mean (SD), mmHg			
Systolic	135 (25)	134 (22)	0.58
Diastolic	84 (15)	85 (14)	0.42
Heart rate, mean (SD), beats/min	74 (17)	77 (16)	0.21
Anemia**	34 (22.1)	42 (18.7)	0.44
Ischemia time, median (IQR), min	169 (110–245)	152 (109–250)	0.18
Single vessel disease	104 (67.5)	154 (68.4)	0.91
**Infarct related artery**			
Left anterior descending coronary artery	59 (38.3)	87 (38.7)	0.97

Left circumflex coronary artery	27 (17.5)	37 (16.4)	
Right coronary artery	68 (44.2)	101 (44.9)	
Left main	0	0	1.00
**Infarct related artery TIMI flow**			
Pre-intervention grade			
0	92 (59.7)	116 (51.6)	0.44
1	11 (7.1)	16 (7.1)	
2	23 (14.9)	43 (19.1)	
3	28 (18.2)	50 (22.2)	
Post-intervention grade			
2	20 (13.0)	14 (6.2)	0.03
3	134 (87.0)	211 (93.8)	
Myocardial blush grade			
0	4 (2.6)	6 (2.7)	0.29
1	16 (10.5)	13 (5.8)	
2	33 (21.6)	41 (18.4)	
3	100 (65.4)	163 (73.1)	
**Procedural characteristics**			
Use of Ioxaglate†	151 (99.3)	222 (99.6)	1.00
Contrast dose, median (IQR), ml	150 (120–185)	140 (120–175)	0.38
Length of procedure, median (IQR), min	32 (24–44)	29 (22–40)	0.08
Radiation exposure, median (IQR), μGy/m^2	5465 (3369–7375)	4837 (2816–7655)	0.18
**Laboratory values at admission**			
Creatinine, mean (SD), µmol/L	73 (16)	73 (15)	0.59
eGFR‡, mean (SD), ml/min/1.73m^2	91 (17)	93 (15)	0.20
CK, median (IQR), U/L	16 (12–27)	16 (13–23)	0.53
Myocardial band of CK, median (IQR), U/L	113 (48–257)	67 (35–156)	<0.01

NT-proBNP, median (IQR), ng/L	8.3 (7.0–9.6)	8.2 (7.0–9.7)	0.80
Glucose, median (IQR), mmol/L	5.8 (5.6–6.1)	5.8 (5.6–6.0)	0.24
HbA_{1C}, median (IQR), %	9 (1)	9 (1)	0.17
Hb, mean (SD), mmol/L	73 (16)	73 (15)	0.59
**AUC from baseline up to 24 hours**			
CK, median (IQR)	$8.9x10^7$ ($4.0x10^7$–$19.1 x10^7$)	$6.1x10^7$ ($2.3x10^7$–$14.2 x10^7$)	<0.01
Myocardial band of CK, median (IQR)	$9.5x10^6$ ($4.2x10^6$–$18.2x10^6$)	$7.3x10^6$ ($2.7x10^6$–$15.3x10^6$)	0.03

CI-AKI contrast-induced acute kidney injury, *SD* standard deviation, *PCI* percutaneous coronary intervention, *IQR* interquartile range, *TIMI* thrombolysis in myocardial infarction, *CAG* coronary arteriography, *eGFR* estimated glomerular filtration rate, *CK* creatine kinase, *NT-proBNP* N-terminal pro–B-type natriuretic peptide, HbA_{1C} glycated hemoglobin, *Hb* hemoglobin, *AUC* area under the curve.

*Calculated as weight in kilograms, divided by lenght in meters squared.
**Defined as Hb<13.7 mg/dl (<8.5 mmol/L) in men and Hb<12.1 mg/dl (<7.5 mmol/L) in women.
†Type of contrast agent was known for 375 (98.9%) patients.
‡Calculated using the Chronic Kidney Disease Epidemiology Collaboration study equation.

Online Resource 3 – Baseline characteristics

Characteristic	No. (%) Total (n = 379)	Metformin (n = 191)	Placebo (n = 188)
Age at randomization, mean (SD), years	58.8 (11.6)	58.7 (11.8)	58.8 (11.5)
Women	95 (25.1)	47 (24.6)	48 (25.5)
Body mass index*, mean (SD), kg/m^2	27.0 (3.8)	26.9 (3.8)	27.0 (3.9)
Race/ethnicity			
White	365 (96.3)	185 (96.9)	180 (95.7)
Asian	10 (2.6)	5 (2.6)	5 (2.7)
Black	4 (1.1)	1 (0.5)	3 (1.6)
**Cardiovascular related history**			
Hypertension	112 (30.0)	61 (31.9)	51 (27.1)
Dyslipidemia	239 (63.1)	111 (58.1)	128 (68.1)
Current smoking	209 (55.1)	108 (56.5)	101 (53.7)
Stroke	3 (0.8)	2 (1.0)	1 (0.5)
Peripheral artery disease	0	0	0
Previous PCI	4(1.1)	1 (0.5)	3 (1.6)
Blood pressure, mean (SD), mmHg			
Systolic	134 (23)	135 (23)	134 (24)
Diastolic	84 (15)	85 (14)	84 (15)
Heart rate, mean (SD), beats/min	76 (16)	75 (16)	77 (16)
Anemia**	76 (20.1)	43 (22.9)	33 (17.3)
Ischemia time, median (IQR), min	161 (109–250)	171 (110–272)	153 (108–234)
Single vessel disease	258 (68.1)	122 (63.0)	136 (72.3)
**Infarct related artery**			
Left anterior descending coronary artery	146 (38.5)	75 (39.3)	71 (37.8)
Left circumflex coronary artery	64 (16.9)	33 (17.3)	31 (16.5)
Right coronary artery	169 (44.6)	83 (43.5)	86 (45.7)
Left main	0	0	0

**Infarct related artery TIMI flow**			
Pre-intervention grade			
0	208 (54.9)	99 (51.8)	109 (58.0)
1	27 (7.1)	14 (7.3)	13 (6.9)
2	66 (17.4)	45 (23.6)	21 (11.2)
3	78 (20.6)	33 (17.3)	45 (23.9)
Post-intervention grade			
2	34 (9.0)	24 (12.6)	10 (5.3)
3	345 (91.0)	167 (87.4)	178 (94.7)
Myocardial blush grade			
0	10 (2.6)	6 (3.2)	4 (2.1)
1	29 (7.7)	20 (10.6)	9 (4.8)
2	74 (19.5)	35 (18.6)	39 (20.7)
3	263 (69.4)	127 (67.6)	136 (72.3)
**Procedural characteristics**			
Use of Ioxaglate†	373 (99.5)	185 (99.5)	188 (99.5)
Contrast dose, median (IQR), ml	150 (120–180)	150 (120–180)	140 (110–176)
Length of procedure, median (IQR), min	31 (22–42)	29 (22–42)	32 (23–42)
Radiation exposure, median (IQR), µGy/m^2	5130 (2944-7646)	5203 (3419–7881)	4895 (2715–7032)
**Laboratory values at admission**			
Creatinine, mean (SD), µmol/L	73 (15)	73 (16)	73 (15)
eGFR‡, mean (SD), ml/min/1.73m^2	92 (16)	93 (17)	93 (14.4)
CK, median (IQR), U/L	129 (83-210)	133 (87–260)	123 (82–181)
Myocardial band of CK, median (IQR), U/L	16 (13-25)	16 (13–29)	16 (12–23)
NT-proBNP, median (IQR), ng/L	81 (40-200)	83 (41–235)	79 (38–176)
Glucose, median (IQR), mmol/L	8.2 (7.0-9.6)	8.2 (7.0–9.4)	8.4 (7.2–9.8)
HbA_{1C}, median (IQR), %	5.8 (5.6-6.0)	5.8 (5.6–6.1)	5.8 (5.6–6.0)
Hb, mean (SD), mmol/L	8.9 (0.8)	9.0 (0.8)	8.9 (0.9)

**AUC from baseline up to 24 hours**			
CK, median (IQR)	$7.1x10^7$ ($3.0x10^7$–$15.1x10^7$)	$7.0x10^7$ ($2.8x10^7$–$15.4\ x10^7$)	$7.1x10^7$ ($3.1x10^7$–$15.1\ x10^7$)
Myocardial band of CK, median (IQR)	$8.2x10^6$ ($3.4x10^6$–$15.8\ x10^6$)	$7.6x10^6$ ($3.3x10^6$–$16.8x10^6$)	$8.7x10^6$ ($3.5x10^6$–$15.3x10^6$)

CI-AKI contrast-induced acute kidney injury, *SD* standard deviation, *PCI* percutaneous coronary intervention, *IQR* interquartile range, *TIMI* thrombolysis in myocardial infarction, *CAG* coronary arteriography, *eGFR* estimated glomerular filtration rate, *CK* creatine kinase, *NT-proBNP* N-terminal pro–B-type natriuretic peptide, HbA_{1C} glycated hemoglobin, *Hb* hemoglobin, *AUC* area under the curve.

*Calculated as weight in kilograms, divided by lenght in meters squared.
**Defined as Hb<13.7 mg/dl (<8.5 mmol/L) in men and Hb<12.1 mg/dl (<7.5 mmol/L) in women.
†Type of contrast agent was known for 375 (98.9%) patients.
‡Calculated using the Chronic Kidney Disease Epidemiology Collaboration study equation.

Online Resource 4 – Adjusted renal function and differences in renal function at individual time points

	eGFR*					Difference in eGFR**		
	N	Metformin	N	Placebo	*p†*	Metformin	Placebo	*p†*
Baseline	188	92 (0.8)	191	92 (0.8)	0.81			
6 hours	186	93 (0.8)	186	94 (0.8)	0.43	1.4 (0.6)	2.0 (0.6)	0.43
12 hours	184	91 (0.8)	181	92 (0.9)	0.40	-0.5 (0.6)	0.2 (0.6)	0.40
24 hours	151	89 (0.9)	146	89 (0.9)	0.91	-2.7 (0.6)	-2.9 (0.6)	0.86
48 hours	82	85 (1.0)	72	84 (1.0)	0.71	-7.2 (0.8)	-8.0 (0.8)	0.47
2 weeks	175	84 (0.9)	171	85 (0.9)	0.38	-7.7 (0.6)	-7.0 (0.6)	0.38
6 weeks	171	86 (0.9)	165	85 (0.9)	0.40	-6.0 (0.6)	-7.3 (0.6)	0.14
4 months	164	86 (1.0)	169	85 (1.0)	0.49	-5.9 (0.8)	-7.1 (0.8)	0.26

Values are expressed as mean (standard error of the mean). Estimated glomerular filtration rate (eGFR) was adjusted using a mixed-effects repeated measurements model for age, baseline N-terminal pro–B-type natriuretic peptide concentration, and treatment allocation. We assumed an unstructured covariance structure among serial observations.

* Calculated using the Chronic Kidney Disease Epidemiology Collaboration study equation.
** After adjustment for baseline eGFR.
† Determined using the Student's *t*-test.

Online Resource 5 – Unadjusted renal function and differences in renal function at individual time points

	eGFR*					Difference in eGFR**		
	N	Metformin	N	Placebo	*p*†	Metformin	Placebo	*P*†
Baseline	188	92 (1.2)	191	92 (1.0)	0.83			
6 hours	186	93 (1.1)	186	94 (1.0)	0.62	1.4 (0.5)	2.1 (0.5)	0.33
12 hours	184	91 (1.2)	181	92 (1.1)	0.60	-0.3 (0.7)	0.2 (0.6)	0.54
24 hours	151	89 (1.3)	146	89 (1.3)	0.79	-2.7 (0.8)	-2.9 (0.8)	0.89
48 hours	82	84 (1.9)	72	82 (1.8)	0.41	-7.6 (1.3)	-8.2 (1.1)	0.72
2 weeks	175	85 (1.3)	171	85 (1.2)	0.74	-7.9 (0.8)	-6.9 (0.7)	0.30
6 weeks	171	87 (1.2)	165	85 (1.2)	0.27	-6.2 (0.8)	-7.5 (0.7)	0.22
4 months	164	86 (1.3)	169	85 (1.2)	0.46	-5.9 (1.0)	-7.1 (0.7)	0.31

Values are expressed as mean (standard error of the mean). *eGFR* estimated glomerular filtration rate.

* Calculated using the Chronic Kidney Disease Epidemiology Collaboration study equation.
** After adjustment for baseline eGFR.
† Determined using the Student's *t*-test.

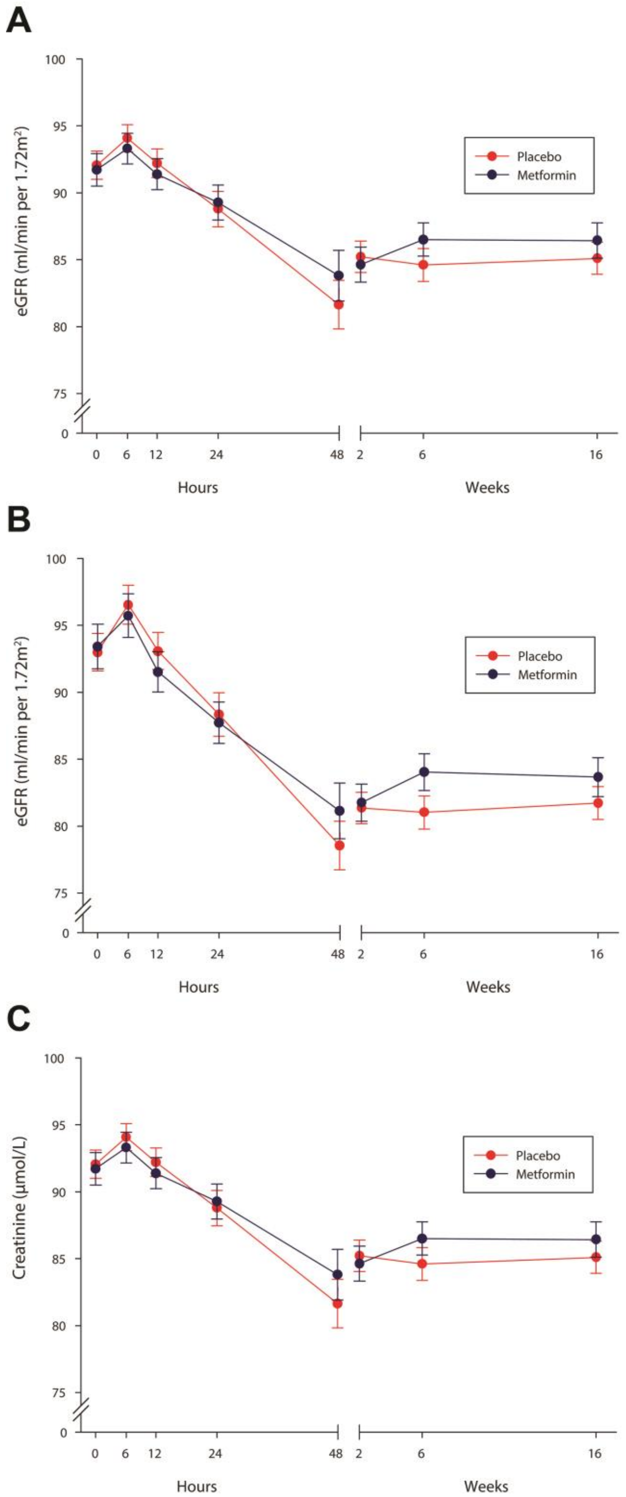

Online Resource 6 - Unadjusted renal function from baseline up to 4 months after primary percutaneous coronary intervention

Values are expressed as mean ± standard error of the mean. Renal function is assessed using **a** the estimated glomerular filtration rate (eGFR) calculated using the Chronic Kidney Disease Epidemiology Collaboration study equation, **b** the eGFR calculated using the Modification of Diet in Renal Disease study equation, and **c** serum creatinine concentration.

Online Resource 7 – Medical therapy at admission

**Drug category**	Total (n=379)	CI-AKI (n=56)	No CI-AKI (n=323)	P-value
Aspirin	26 (6.9)	4 (7.1)	22 (6.7)	1.00
Coumarin derivative	2 (0.5)	0	2 (0.6)	1.00
Thienopyridines	2 (0.5)	0	2 (0.6)	1.00
Clopidogrel	2 (0.5)	0	2 (0.6)	1.00
Prasugrel	0	0	0	
Ticagrelor	0	0	0	
ACE-inhibitor or ARB	46 (12.1)	7 (12.5)	39 (12.1)	1.00
Beta-blocker	41 (10.8)	5 (8.9)	36 (11.1)	0.82
Calcium-channel blocker	18 (4.7)	6 (10.7)	12 (3.7)	0.04
Mineralocorticoid receptor antagonist	2 (0.5)	0	2 (0.6)	1.00
Other diuretic	36 (9.5)	6 (10.7)	30 (9.3)	0.80
Statin	34 (9.0)	7 (12.5)	27 (8.4)	0.31

Values are expressed as no (%). *ARB* angiotensin-receptor blocker.

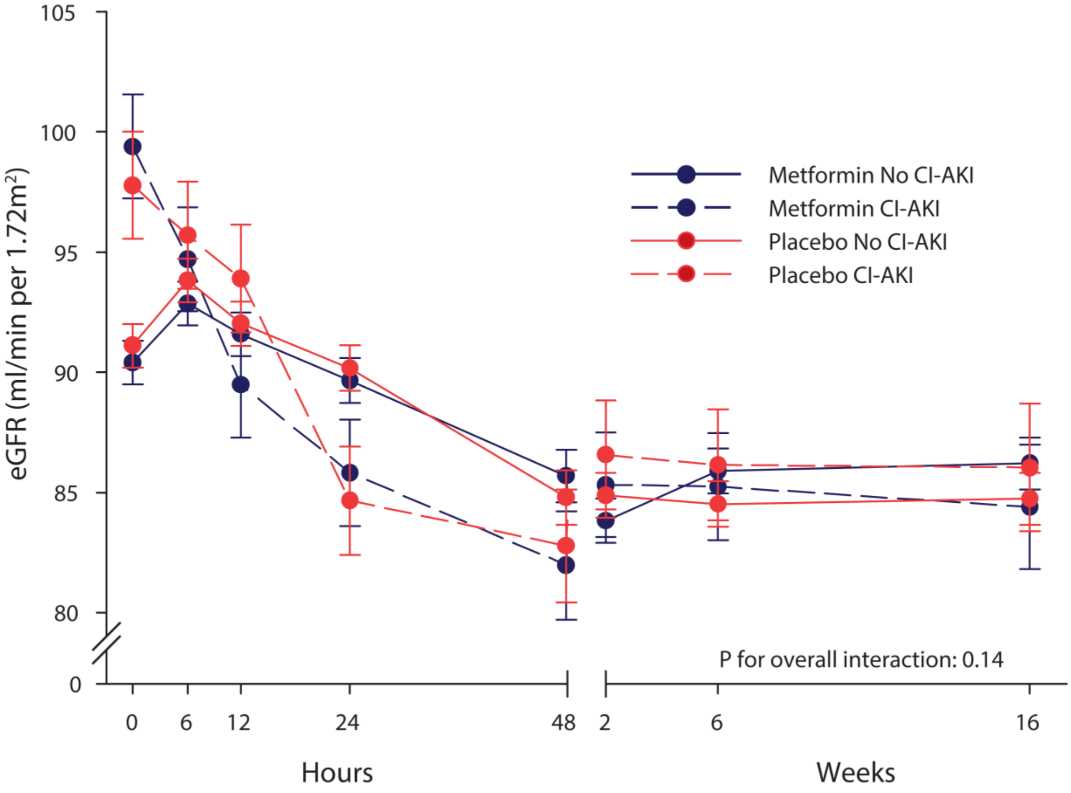

Online Resource 8 – Presented are least-squares means ± standard error from the mixed-effects repeated measurements model with a random intercept and slope. Individual patients were considered as random effects and the following were fixed effects: age, baseline N-terminal pro–B-type natriuretic peptide concentration, development of CI-AKI, and treatment allocation. We assumed an unstructured covariance structure among serial estimated glomerular filtration rate values (eGFR). No significant difference was observed for the overall interaction of CI-AKI, time and allocated treatment (*P*=0.14). There was an expected overall interaction between the occurrence of CI-AKI and the change in eGFR over the first 48 hours (P=0.021), but there was no difference between randomized treatments (P=0.13). At the end of follow up, eGFR did not differ between patients on placebo or metformin, neither in the CI-AKI group (P=0.34) nor the no CI-AKI group (P=0.66).
